# Laquinimod efficacy in relapsing-remitting multiple sclerosis: how to understand why and if studies disagree

**DOI:** 10.1186/s12883-016-0702-4

**Published:** 2016-09-17

**Authors:** Gary R. Cutter, Volker Knappertz, Nissim Sasson, David Ladkani

**Affiliations:** 1 Department of Biostatistics, School of Public Health, University of Alabama at Birmingham, Room 327, 1720 2nd Avenue South, Birmingham, AL 35294 USA; 2 Department of Neurology and Psychiatry, Heinrich-Heine University, Düsseldorf, Germany; 3 Teva Pharmaceutical Industries, Frazer, PA USA; 4 Teva Pharmaceutical Industries Abic, Ltd, Netanya, Israel; 5 Teva Pharmaceutical Industries, Petach Tikva, Israel

**Keywords:** Propensity score, Interferon beta-1b, Laquinimod, Relapsing-remitting multiple sclerosis

## Abstract

**Background:**

The results of two randomized phase 3 trials that investigated the use of laquinimod in patients with relapsing-remitting multiple sclerosis were analyzed using a propensity score model.

**Methods:**

The propensity score in each study was defined as the probability of an individual patient being assigned to either the laquinimod or placebo study arm. The analysis included two main stages: (1) calculation of a propensity score for each patient, given a broad set of baseline covariates that included second-degree interactions, and (2) incorporation of the propensity score as another covariate into the predefined primary analysis model to test the treatment effect of laquinimod (0.6 mg/d) vs placebo on the annualized relapse rate (ARR).

**Results:**

The BRAVO study showed baseline imbalances for T2 volume and the proportion of patients with gadolinium (Gd)-enhancing lesions, both parameters known to correlate with risk of relapse. Adjustment using the propensity score as a categorical variable showed that the estimated difference in ARR between laquinimod and placebo was 0.078, in favor of laquinimod.

In ALLEGRO, the baseline Gd-enhancing lesion mean score was higher for placebo vs laquinimod. When the primary analysis model was adjusted for the propensity score as a categorical variable, the covariate adjusted difference in mean ARR between laquinimod and placebo was 0.084, in favor of laquinimod.

**Conclusions:**

Propensity scores addressing differences in baseline characteristics may be helpful to better understand whether observed treatment effect differences in randomized controlled trials are accurate results or result from inherent differences between patients with multiple sclerosis.

## Background

The propensity score has become increasingly popular for adjusting uncontrolled treatment assignment in observational studies [1–7]. Absent randomization, baseline characteristics may differ markedly between groups, which may affect the validity of study findings [3–5]. In recent years, propensity scores have been used to adjust for covariate imbalance in nonrandomized studies [8–10] and randomized controlled trials [11–13] in numerous therapeutic areas, including multiple sclerosis (MS).

The purpose of the current study was to use a propensity score model to reexamine the results of 2 randomized phase 3 trials that investigated the use of laquinimod in patients with relapsing-remitting MS (RRMS): Assessment of Oral Laquinimod in Preventing Progression in Multiple Sclerosis (ALLEGRO) and Benefit-Risk Assessment of Avonex and Laquinimod (BRAVO). Although the study designs of ALLEGRO and BRAVO were similar and were conducted in roughly the same calendar years, the treatment effect of laquinimod on the annualized relapse rate (ARR) and disability progression was found, based upon a predefined analysis plan, to be more favorable in the former than in the latter trial [14, 15]. Subsequent investigations, including a reexamination of the results of a prespecified sensitivity analysis and further *post hoc* sensitivity analyses, suggested that there was imbalance in a fundamental baseline magnetic resonance imaging (MRI) characteristic known to impact clinical outcomes. We used a propensity score model to reassess the study results and strengthen the findings of previous analyses by establishing more consistency via understanding of the imbalances.

## Methods

The results of the ALLEGRO and BRAVO trials have been described elsewhere, as have the findings of the prespecified and *post hoc* sensitivity analyses [14, 15]. The following summaries are provided as context for the current propensity score analysis.

### Main findings from ALLEGRO and BRAVO

ALLEGRO was a double-blind, international study in 1106 patients with RRMS who were randomly assigned in a 1:1 ratio to receive either oral laquinimod 0.6 mg once daily or oral placebo for 24 months [14]. Treatment with laquinimod vs placebo was associated with a reduction in the mean ± standard error (SE) ARR (0.30 ± 0.02 for laquinimod vs 0.39 ± 0.03, for placebo, *P* = 0.002). Laquinimod was also associated with significant reductions in the risk of 3-month confirmed disability progression, the mean cumulative number of gadolinium (Gd)-enhancing lesions at 12 and 24 months, and the cumulative number of new or enlarging lesions on T2-weighted images [14].

BRAVO was a placebo-controlled, international study in 1331 patients with RRMS who were randomly assigned with equal probability to receive oral laquinimod 0.6 mg once daily, matching oral placebo, or interferon beta-1a (IFNβ-1a) (30 μg intramuscularly once weekly) for 24 months [15]. Patients who received IFNβ-1a were excluded from this analysis, which is simplified with just two groups. Treatment with laquinimod vs placebo was associated with a nonsignificant reduction in ARR (0.28 ± 0.03 for laquinimod vs 0.34 ± 0.03 for placebo; risk ratio [RR] 0.82; 95 % confidence interval [CI] 0.66–1.02; *P* = 0.075) [15]. Percent brain volume change from baseline to month 24 was significantly reduced with laquinimod vs placebo [15].

### Findings from prespecified and *post hoc* analyses

The BRAVO prespecified sensitivity analysis revealed that the baseline mean volume of T2 lesions was greater for laquinimod (9.6 cm^3^) than for placebo (7.9 cm^3^, *P* = 0.009). Further, more patients in the laquinimod group (40 %) had Gd-enhancing lesions at baseline, despite randomization, than did those in the placebo group (33 %, *P* = 0.055) [15]. Previous literature has shown that the number of new, active T2 lesions can serve as a predictor of rate of relapse both in individual-patient analysis and as observed in the ratio between experimental and control arms in studies [16, 17]. Similarly, the proportion of patients with Gd-enhancing lesions and T2 lesion volume at baseline was found to be a strong predictor of the rate of relapse during the BRAVO study (β linear estimates of 0.45 with *P* < 0.0001 for the categorical Gd-enhancing T1 lesions and 0.0112 with *P* = 0.0126 for the continuous T2 volume variables); therefore, they were added as covariates to the statistical model for the purpose of conducting several *post hoc* analyses [15].

In one *post hoc* analysis of the BRAVO study that included the two baseline MRI parameters as covariates, the ARR for laquinimod vs placebo was reduced by 21 % (*P* = 0.0264), and the risk of worsening of disability confirmed at 3 months for laquinimod vs placebo was reduced by 33.5 % (*P* = 0.044) [15]. In another *post hoc* analysis of the BRAVO study, the observed relapse rate in the placebo group at 24 months was found to be lower (0.34 relapses/year) than expected (0.6 relapses/year) based on a *post hoc* power calculation made for the study design, and thus, had the study been conducted with this knowledge, it would have had only 48 % statistical power to detect a significant treatment of the observed effect of laquinimod vs placebo on ARR [15].

### Propensity score model

The results from BRAVO suggested that, although randomization assigned treatments in an unbiased manner, imbalances still occurred, and exploring these might improve the understanding of the results. Thus, exploration via propensity scores might be useful. The *propensity score* was defined as the probability of an individual patient being assigned to either of the study arms (laquinimod or placebo) given a known set of covariates. If balance is to be obtained in those covariates, it is expected that for treatment the propensity score would revolve around 0.5 (given 2 treatment groups). The propensity to be allocated into each group was summarized into 1 score, and that score was used as a covariate with 1 degree of freedom (in the case of a continuous covariate) in the primary analysis model; this method allows for adjustment as compared with performing analysis of covariance, which may involve too many covariates simultaneously.

The goal of using a propensity score was to obtain an estimate of the probability of being assigned to 1 or another of the treatment arms based on characteristics within the trial, when the theoretical probability was known to be 0.50 [3]. A major concern in the use of propensity score analyses is having unmeasured covariates critical in the assignment of treatments. This is not the concern here because we accept that randomization has balanced the unmeasured covariates and we are only adjusting for known differences as explanations for differences in results. However, the potential for unmeasured confounding variables cannot be fully ruled out, and it may represent a potential limitation of the study. Independent variables included pretreatment covariates that may have been associated with treatment imbalance, as well as the reported number of relapses. Explanatory variables included age, sex, country, weight at baseline, time from first symptom, time from diagnosis, tobacco use, indicator for the number of Gd-enhancing lesions at baseline, log of the total number of exacerbations in the last year, log of the total number of exacerbations in the last 2 years, baseline Expanded Disability Status Scale (EDSS) score, baseline Multiple Sclerosis Functional Composite score, T2 lesion volume at baseline, T1 lesion hypointense volume at baseline, and normalized brain volume at baseline. All second-degree interactions with the variables listed were also included in the model, with the exception of interactions with country because of the small number of patients from some countries.

Baseline covariates not included because of missing values were as follows: EDSS score on date of onset of last exacerbation prior to randomization, EDSS score on date of diagnosis of MS, time from date of onset of last exacerbation, time from stabilization of last exacerbation, and previous exposure to glatiramer acetate; race was also omitted because nearly all of the patients were white.

The analysis included two main stages: (1) calculation of a propensity score for each patient, given a broad set of baseline covariates that also included second-degree interactions, and then (2) incorporating the propensity score as another covariate into the predefined primary analysis model to test the treatment effect of laquinimod (0.6 mg/d) vs placebo on ARR. For comparative purposes of this approach, the latter stage used two adjustments approaches: one included a continuous propensity score as a covariate, and the other subclassified the range of the continuous propensity scores into quintiles and included the quintile as a categorical variable (with 5 levels and 4 degrees of freedom).

### Statistical analyses

The logistic regression model estimated the probability for each patient to be assigned to the laquinimod arm; thus, as expected from the baseline imbalances, propensity scores for patients in both BRAVO and ALLEGRO who actually received laquinimod were lower than those for patients who actually received placebo. To simplify the presentation of results, quintiles were used as categorical variables in the current analysis [18]. Propensity score quintiles were calculated by combining the range of values in the laquinimod and placebo groups.

## Results

### BRAVO study

In total, 880 patients were included in the BRAVO propensity score analysis; of these, 431 (of 434) were in the laquinimod arm and 449 (of 450) were in the placebo arm. Four patients (3 from the laquinimod arm and 1 from the placebo arm) had missing values in Gd-enhancing lesions and were not included in the analysis.

Summary statistics of propensity scores by treatment group are shown in Table 1.

**Table 1:** Distribution of propensity score by treatment group in the BRAVO study

	Treatment group	
	Placebo	Laquinimod 0.6 mg/day
	Estimate	Estimate
Quartile		
Maximum		
Upper quartile	0.701	0.529
Median	0.586	0.407
Lower quartile	0.440	0.267
Minimum	0.109	0.007

**Fig. 1:**
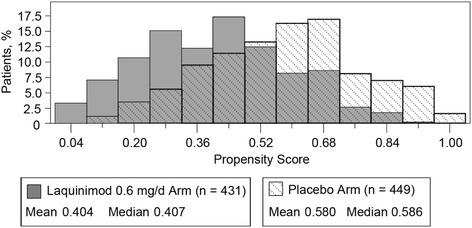
Double-blind intent-to-treat distribution of propensity scores by treatment group in the BRAVO study

The distribution of propensity scores by treatment group is shown in Fig. 1. The shift in the distribution between the groups is consistent with the slight imbalances seen in the baseline characteristics. As shown, the shift of the propensity score distributions between placebo and laquinimod suggests higher propensity (given the set of covariates) to be allocated to placebo rather than laquinimod, had the randomization been performed as a function of the covariates included. Individual propensity scores by quintile are shown in Table 2.

**Table 2:** Subclasses of propensity score in the BRAVO study

Subclass number	Quintile range
1	0.00653–0.29832
2	0.29832–0.42971
3	0.42971–0.54541
4	0.54541–0.67484
5	0.67484–0.99074

In the unadjusted primary analysis of the BRAVO study, laquinimod reduced the risk of relapse by 18 % vs placebo (RR = 0.82; 95 % CI 0.66–1.02; *P* = 0.075), and the unadjusted mean ARR was 0.28 ± 0.03 for laquinimod versus 0.34 ± 0.03 for placebo, a difference of 0.06 [15]. The primary analysis model for the effect on ARR adjusting for continuous propensity score revealed that laquinimod reduced the risk of relapse by 23.1 % vs placebo (RR = 0.769; 95 % CI 0.610–0.969; *P* = 0.026). The estimated mean ARR was 0.268 (95 % CI 0.222–0.324) for laquinimod vs 0.349 (95 % CI 0.292–0.417) for placebo, a difference of 0.081. Adjustment using the propensity score as a categorical variable showed that laquinimod similarly reduced the risk of relapse by 22.4 % vs placebo (RR = 0.776; 95 % CI 0.616–0.978; *P* = 0.0315), and the estimated ARR was 0.267 (95 % CI 0.221–0.323) in the laquinimod group and 0.345 (95 % CI 0.288–0.412) in the placebo group, a difference of 0.078.

### ALLEGRO study

In total, 1096 patients were included in the ALLEGRO propensity score analysis; of these, 543 (of 550) were in the laquinimod arm and 553 (of 556) were in the placebo arm. Ten patients (7 from the laquinimod arm and 3 from the placebo arm) had missing values (that is, brain volume, Gd-enhancing lesions, T2 volume, T1 hypointense volume, weight, body mass index) and were not included in the analysis.

Summary statistics of propensity scores by treatment group are shown in Table 3.

**Table 3:** Distribution of propensity score by treatment group in the ALLEGRO study

	Treatment group	
	Placebo	Laquinimod 0.6 mg/day
	Estimate	Estimate
Quartile		
Maximum		
Upper quartile	0.647	0.547
Median	0.540	0.448
Lower quartile	0.445	0.339
Minimum	0.106	0.034

**Fig. 2:**
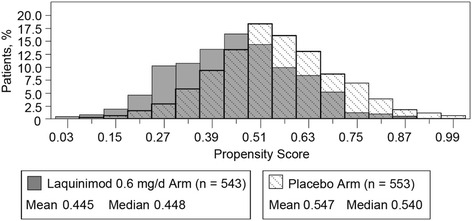
Double-blind intent-to-treat distribution of propensity scores by treatment group in the ALLEGRO study

The distribution of propensity scores by treatment group is shown in Fig. 2. As can be seen, the shift of the propensity score distributions between placebo and laquinimod suggests higher propensity (given the set of covariates) to be allocated to placebo rather than laquinimod. Propensity scores by quintile are shown in Table 4.

**Table 4:** Subclasses of propensity score in the ALLEGRO study

Subclass number	Quintile range
1	0.033837–0.36007
2	0.36007–0.46234
3	0.46234–0.52988
4	0.52988–0.63189
5	0.63189–0.99533

In the unadjusted primary analysis of the ALLEGRO study, laquinimod reduced the risk of relapse by 23 % vs placebo (RR = 0.77; 95 % CI 0.65–0.91; *P* = 0.002), and the unadjusted mean ARR was 0.30 ± 0.02 for laquinimod versus 0.39 ± 0.03 for placebo, a difference of 0.09 [14]. When the primary analysis model was adjusted for the propensity score as a continuous covariate, laquinimod reduced the risk of relapse by 19.2 % vs placebo (RR = 0.808; 95 % CI 0.675–0.967; *P* = 0.0201), and the covariate adjusted mean ARR was 0.308 (95 % CI 0.265–0.357) for laquinimod vs 0.381 (95 % CI 0.332–0.437) for placebo, a difference of 0.073. When the primary analysis model was adjusted for the propensity score as a categorical variable, laquinimod reduced the risk of relapse by 21.7 % vs placebo (RR = 0.783; 95 % CI 0.655–0.936; *P* = 0.0073), and the covariate adjusted mean ARR was 0.303 (95 % CI 0.261–0.351) for laquinimod and 0.387 (95 % CI 0.337–0.444) for placebo, a difference of 0.084.

## Discussion

When a randomized trial is analyzed, the pre-planned analyses using intention-to-treat or modified intention-to-treat remain the preferred approach and the first analysis to be done. However, when parallel trials are conducted and differences in results are observed, it is important to understand whether the divergent results are caused by a lack of consistency in the outcomes or whether the differences can be explained by the impact of covariates that were measured. The results of the current *post hoc* sensitivity analysis using propensity scores in the reanalysis of data from the ALLEGRO and BRAVO studies demonstrate that, although randomization guarantees unbiased assignment, randomization does not guarantee equality. Often the variations in covariates that exist are still the result of unbiased assignment but may lead to observed differences in covariates that can, and do, have measurable effects on trial outcomes. In this example, the original preplanned analysis was most likely affected by imbalances in important baseline characteristics, and the propensity-adjusted analyses confirm that the estimate of the treatment effect is quite similar.

The propensity score, which can capture potential imbalances concealed in baseline covariates in a single number, can be implemented to strengthen a covariate sensitivity analysis without overfitting a model by using too many covariates. Propensity scores may be included in statistical analyses through matching, stratification, or regression adjustment; however, these analyses are most often included in a regression model as an explanatory variable, which is tantamount to covariate adjustment, but sparing the degrees of freedom [19].

The BRAVO study showed baseline imbalance with regard to T2 volume and the proportion of patients with Gd-enhancing lesions. Both parameters are known to be correlated with the occurrence of relapses, thereby having a potential impact on the observed treatment effect of laquinimod on ARR. When both baseline MRI parameters were introduced into the BRAVO primary model, they were found to be strong predictors of the relapse rate during the double-blind treatment phase; a similar correlation was found in the ALLEGRO study. Unlike in BRAVO, the inclusion of the propensity score in ALLEGRO decreased the treatment effect. The linear estimate of the relationship between propensity score and treatment effect was negative in ALLEGRO and positive in BRAVO (when the propensity is defined as the probability to be assigned with laquinimod), which explains the opposite influence on the treatment effect, while trends in propensity as shown in Figs. 1 and 2 are similar.

Numerous examples of the use of propensity scores to analyze data from randomized controlled trials (RCTs) can be found in the literature, and one study is of particular interest regarding the current discussion.

Recently, two different research groups conducted *post hoc* analyses of the Atrial Fibrillation Follow-up Investigation of Rhythm Management (AFFIRM) trial to evaluate the causal effects of digoxin treatment on mortality in patients with recurrent persistent atrial fibrillation who were randomized to rate-control or rhythm-control strategies [13, 20]. Both research groups used AFFIRM data, and both groups used propensity score analysis (albeit different approaches), but their findings differed [13]. Researchers who used propensity score covariate adjustment reported that digoxin was associated with an increase in all-cause mortality, whereas researchers who used propensity score matching reported that it was not [21, 22]. Although the use of different propensity score approaches could potentially account for the discrepancy, other explanations, including differences in patient selection and the classification of digoxin exposure, are also possible [13].

RCTs are viewed as the “gold standard” in clinical trial design because, in general, they generate standardized data from patient subgroups whose selection has been based on strict criteria [2]. Indeed, one perspective is that if baseline randomization is performed accurately, different outcomes among patient subgroups represent a treatment effect [2, 23]. In the case of unexpected findings from RCTs or the suspicion of covariate imbalance, statistical reanalysis using propensity scores may be appropriate to better align patient subgroups when assessing significance of treatment.

## Conclusions

The current propensity analysis demonstrates through example that baseline imbalances in RCTs such as those that occurred in BRAVO, which were absent in ALLEGRO, can contribute to numerical differences in treatment results. When adjusted and better aligned, the results of BRAVO more closely resembled those of ALLEGRO. These results point out that randomized studies that may seem slightly inconsistent may, in fact, be quite similar, and propensity score analyses might enable identification of those studies that are consistent with each other and those that are not. Specifically, in the MS treatment paradigm of reduction of relapse rates, propensity scores using baseline MRI characteristics can prove helpful to adjust for and better reflect observed differences in treatment effect in RCTs.

## Abbreviations

AFFIRM: Atrial Fibrillation Follow-up Investigation of Rhythm Management
ALLEGRO: Assessment of Oral Laquinimod in Preventing Progression in Multiple Sclerosis
ARR: Annualized relapse rate
BRAVO: Benefit-Risk Assessment of Avonex and Laquinimod
CI: Confidence interval
EDSS: Enhanced Disability Status Scale
Gd: Gadolinium
IFNβ-1a: Interferon beta-1a
MRI: Magnetic resonance imaging
MS: Multiple sclerosis
RCT: Randomized controlled trial
RR: Risk of relapse
RRMS: Relapsing-remitting multiple sclerosis
SE: Standard error
